# Effect of silver sulfadiazine on mature mixed bacterial biofilms on voice prostheses

**DOI:** 10.1186/s40463-023-00672-3

**Published:** 2023-11-21

**Authors:** Yanyan Niu, Yongli Zhang, Hong Huo, Xiaofeng Jin, Jian Wang

**Affiliations:** 1 Department of Otolaryngology, Peking Union Medical College Hospital, Peking Union Medical College, Chinese Academy of Medical Sciences, 1# ShuaiFuYuan, Dongcheng District, Beijing, 100730 People’s Republic of China; 2 Department of Otorhinolaryngology Head and Neck Surgery, China-Japan Friendship Hospital, Beijing, People’s Republic of China

**Keywords:** Silver sulfadiazine, Voice prostheses, Mature biofilms, Mixed strains, Removal, Inhibition

## Abstract

**Background:**

Biofilm formation on voice prostheses disrupts the function and limits the lifespan of voice prostheses. There is still no effective clinical strategy for inhibiting or removing these biofilms. Silver sulfadiazine (SSD), as an exogenous antibacterial agent, has been widely used in the prevention and treatment of infection, however, its effect on voice prosthesis biofilms is unknown. The purpose of this study was to explore the effect of SSD on the mature mixed bacterial biofilms present on voice prostheses.

**Methods:**

Quantitative and qualitative methods, including the plate counting method, real-time fluorescence quantitative PCR, crystal violet staining, the 2,3-bis(2-methoxy-4-nitro-5-sulfophenyl)-2H-tetrazolium-5-carboxanilide) (XTT) reduction assay, scanning electron microscopy, and laser confocal microscopy, were used to determine the effect of SSD on the number of bacterial colonies, biofilm formation ability, metabolic activity, and ultrastructure of biofilms in a mature mixed bacterial (*Staphylococcus aureus*, *Streptococcus faecalis* and *Candida albicans*) voice prosthesis biofilm model. The results were verified in vitro on mature mixed bacterial voice prosthesis biofilms from patients, and the possible mechanism of action was explored.

**Results:**

Silver sulfadiazine decreased the number of bacterial colonies on mature mixed bacterial voice prosthesis biofilm, significantly inhibited the biofilm formation ability and metabolic activity of mature voice prosthesis biofilms, inhibited the formation of the complex spatial structure of voice prosthesis biofilms, and inhibited the synthesis of polysaccharides and proteins in the biofilm extracellular matrix. The degree of inhibition and removal effect increased with SSD concentration.

**Conclusions:**

Silver sulfadiazine can effectively inhibit and remove mature mixed bacterial voice prosthesis biofilms and decrease biofilm formation ability and metabolic activity; SSD may exert these effects by inhibiting the synthesis of polysaccharides and proteins among the extracellular polymeric substances of voice prosthesis biofilms.

**Graphical abstract:**

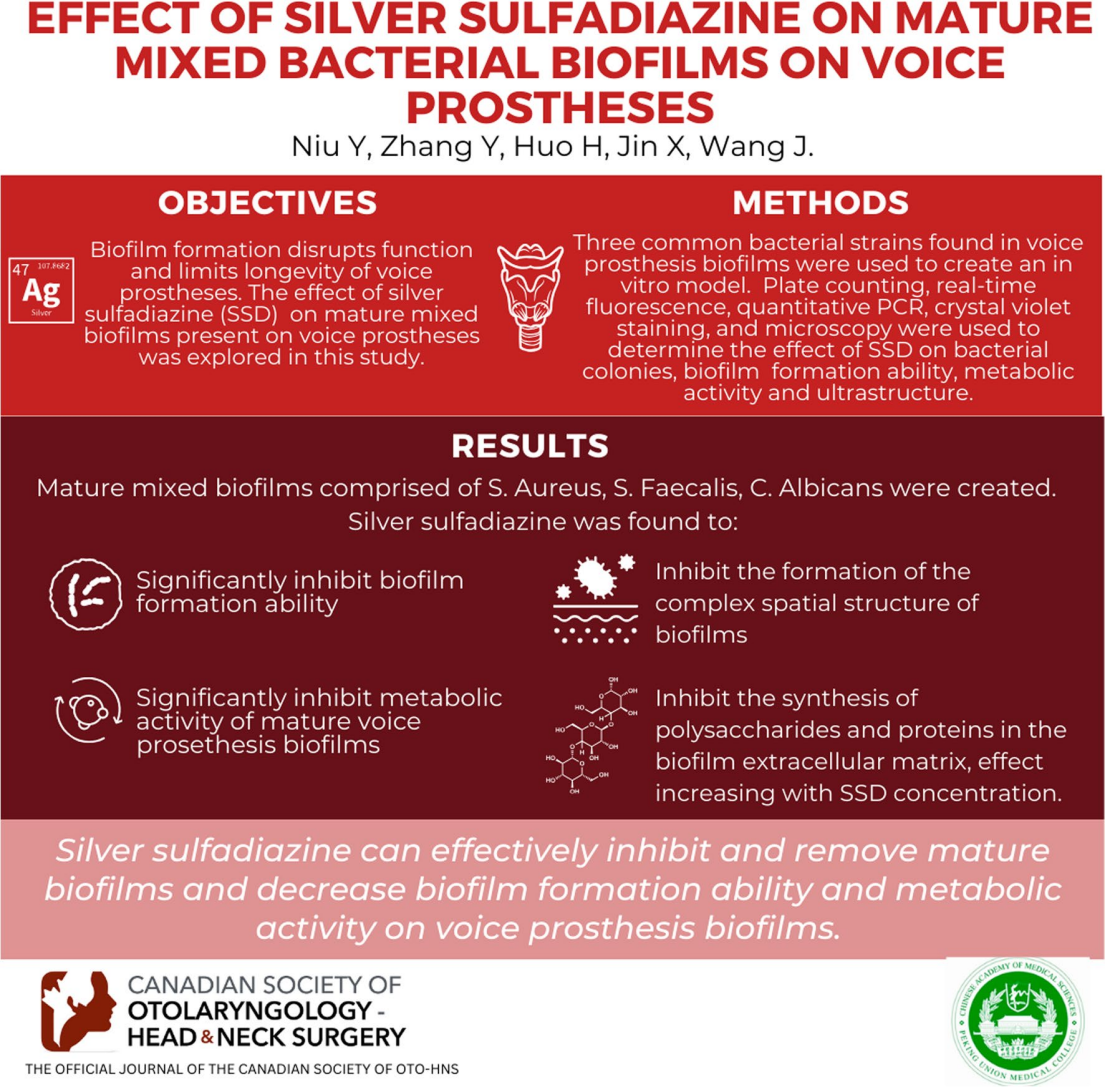

## Background

The loss of voice after total laryngectomy severely affects patients’ personal and social life [1]. As the gold standard for voice rehabilitation after total laryngectomy [2, 3], voice prostheses have significantly improved the quality of life of these patients. Biofilm formation on voice prostheses can lead to leakage of esophageal contents into the airway and increase airflow resistance during speech, which is an important factor affecting the clinical lifetime of voice prostheses. Therefore, voice prostheses need to be replaced every 4–6 months [4]. Furthermore, there are potential complications associated with the replacement of voice prostheses, such as local infection, granuloma formation and replacement failure due to tracheoesophageal fistula stenosis [5]. Although some studies have explored the inhibition of biofilm formation on voice prostheses, no effective method has been developed to prolong the lifespan of these devices in clinical [6].

Silver possesses broad-spectrum antibacterial activity against both multidrug-susceptible and multidrug-resistant strains [7, 8]. Silver ions exert toxicity against microorganisms by affecting respiratory enzymes and components of the microbial electron transport system. In addition, silver ions can bind to bacterial DNA and interfere with transcription and replication processes, thereby exerting a bactericidal effect [9, 10]. To date, various formulations containing silver ions have been shown to be effective at removing bacterial biofilms from nonhealing wounds [11, 12]. As an exogenous antibacterial agent, silver sulfadiazine (SSD) has strong antibacterial effects on gram-positive and gram-negative bacteria, yeast, fungi, and other microorganisms and has been widely used to prevent and treat infections in burn patients [13, 14]. However, the effect of SSD on biofilms and the underlying mechanism are still unclear. Recent studies have used SSD as a layer material for indwelling catheters in vivo [15, 16], but there has been no research on whether SSD can inhibit and remove voice prosthesis biofilms. Based on the reliability and effectiveness of SSD in the clinical treatment and prevention of infection, we hypothesize that SSD may also be able to significantly inhibit and remove mature mixed bacterial biofilms on voice prostheses, thereby providing a novel strategy for prolonging the lifespan of these devices.

In the present study, we investigated the effect of SSD on voice prosthesis biofilms based on a mature mixed bacterial voice prostheses biofilm model. The results were further verified in vitro by using mature mixed bacterial biofilms from patients’ voice prostheses. In addition, we conducted a preliminary exploration of the possible mechanism underlying the effect of SSD on mature voice prosthesis biofilms.

## Methods

### Bacterial strains and materials

In this study, three reported strains commonly found in voice prosthesis biofilms, Staphylococcus aureus (ATCC 25923), Streptococcus faecalis (ATCC 13419) and Candida albicans (SC 5314), were used to construct an in vitro model of voice prosthesis biofilms [17–19]. These three strains were cultured in 70% yeast extract/peptone/dextrose medium (Sigma‒Aldrich) + 30% fetal bovine serum (Gibco) (YPDF medium) and incubated overnight at 37 °C, after which plaques were clearly visible [20]. Medical-grade silicone membranes (thickness: 1 mm) were purchased from Suzhou Shoucheng Electronics Co., Ltd., China, and were sterilized under high pressure at 121 °C before use.

### Construction of mature biofilms on medical silicone membranes

The construction of mature biofilms on medical silicone membranes was carried out as previously reported [20, 21]. Appropriate amounts of mature colonies were picked and cultured in 5 ml of YPDF medium at 37 °C for 6–8 h, until the OD600 was approximately 0.6. These bacterial cultures were diluted and mixed in equal volumes, inoculated into YPDF medium at a 1% ratio, then added to 96-well plates (Corning, 3599) with sterilized medical silicone membranes. The cells were cultured at 37 °C for 48 h.

### Determination of the minimum mature mixed biofilm inhibitory concentration (BIC) and the minimum mature mixed biofilm eradication concentration (BEC) on voice prostheses

Three groups were set up for this experiment, a blank control group (medium only in well, without biofilm or SSD), a negative control group (medium and biofilm in the well, without SSD), and experimental groups (medium, biofilms and SSD at different concentrations). Sterilized medical silicone membranes were placed obliquely in the wells of a 96-well deep-well plate, and 2 ml of YPDF medium was added to each well. Bacterial cultures of each species diluted to 1% were mixed in equal volumes and inoculated into each well. Silver sulfadiazine (Sigma‒Aldrich 481,181-5G) at different concentrations was added, with an equal volume of H_2_O used as a blank control, followed by incubation at 37 °C for 24 h. The number of colonies in each well was measured by the plate counting method. The lowest SSD concentration that reduced the number of colonies in the voice prosthesis biofilms by 50% compared to the blank control was taken as BIC_50_. Compared with the negative control group (SSD concentration: 0 µg/ml), the lowest SSD concentration that reduced the number of colonies in the voice prosthesis biofilms by 30% was taken as BEC_30_, the lowest SSD concentration at which the number of colonies was reduced by 50% was taken as BEC_50_, and the lowest SSD concentration at which the number of colonies was reduced by 70% was taken as BEC_70_.

### Plate counting of biofilms

The biofilms on the medical silicone membranes were eluted by ultrasonication. The bacterial cultures were diluted to different concentrations with phosphate-buffered saline (PBS) (Gibco™, 70011044), and 100 µl of each dilution was spread on a YPDF plate. The cells were incubated at 37 °C for approximately 18 h and counted by taking pictures with an automatic colony counter (Interscience, SCAN1200). The percentage of colonies removed by SSD from the mature voice prosthesis biofilms was calculated by Formula (1):

1 $${\text{Percentage }}\;{\text{of}}\;{\text{ removal}} = \left[ {1 - \frac{{{\text{number }}\;{\text{of }}\;{\text{colonies}}\;{\text{ in}}\;{\text{ experimental}}\;{\text{ group}}}}{{{\text{number}}\;{\text{ of }}\;{\text{colonies}}\;{\text{ in}}\;{\text{ negative}}\;{\text{ control}}\;{\text{ group}}}}} \right] \times 100{\text{\% }}$$

### Real-time fluorescence quantitative PCR

Standard plasmids for *S. aureus*, *S. faecalis* and *C. albicans* were constructed, the concentration of each standard plasmid was adjusted to 10^9^ copies/µl, and each standard plasmid was diluted fivefold to draw a standard curve. A bacterial genome extraction kit (Vazyme, DC103) was used to extract DNA from the bacterial strains in the biofilms, and the SYBR Green Master Mix (Vazyme, Q111-02) kit was used to carry out qPCR on the obtained DNA and plasmids. The final reaction system volume was 10 µl. The reaction conditions were as follows: 50 °C for 2 min and 95 °C for 5 min, followed by 40 cycles of 95 °C for 15 s, 56 °C for 20 s, and 72 °C for 40 s. The real-time fluorescence quantitative PCR primers are shown in Table 1.

**Table 1:** Primers for real-time fluorescence quantitative PCR

Name	Primer	Sequence	Size
ATCC25923	Forward	5'-CAATGGACAATACAAAGGGCAG-3'	82 bp
	Reverse	5'-TGCAGACTACAATCCGAACTG-3'	
ATCC13419	Forward	5'-GTTAGTAACTGAACGTCCCCTG-3'	143 bp
	Reverse	5'-TCAGACTTAAGAAAACCGCCTG-3'	
SC5314	Forward	5'-CTTAAGTTCAGCGGGTAGTCC-3'	140 bp
	Reverse	5'-GAAAGACGGTAGTGGTAAGGC-3'	

In this experiment, a blank control group, a negative control group, and experimental groups (SSD concentrations: BEC_30_, BEC_50_ and BEC_70_ and 5 µg/ml, 10 µg/ml, 15 µg/ml, 20 µg/ml, and 30 µg/ml) were prepared. According to the method described above, a mature mixed bacterial voice prosthesis biofilm was constructed, the medium and different concentrations of SSD were added to the 96-well plate according to the experimental grouping. The cells were cultured at 37 °C for 24 h. Real-time fluorescence quantitative PCR was used to determine the copy numbers of the three strains in the biofilms of each group to evaluate the corresponding inhibitory effects of SSD on the three strains in the biofilms. The percentage of strain copies removed from the biofilms by SSD was calculated by Formula (2).

2 $${\text{Percentage }}\;{\text{of}}\;{\text{ removal}} = \left[ {1 - \frac{{{\text{copy }}\;{\text{number }}\;{\text{of}}\;{\text{ strains}}\;{\text{ in}}\;{\text{ experimental }}\;{\text{group}}}}{{{\text{copy}}\;{\text{ number}}\;{\text{ of }}\;{\text{strains}}\;{\text{ in}}\;{\text{ negative }}\;{\text{control }}\;{\text{group}}}}} \right] \times 100{\text{\% }}$$

### Crystal violet staining

The cultured medical silicone membranes were removed, washed with PBS twice, fixed with 0.10 ml of 10% methanol solution (BBI, A601617) for 15 min, and stained with 0.1% crystal violet staining solution (KeyGEN BioTECH, KGA229) after air-drying. The membranes were then placed at room temperature for 10 min, rinsed three times with PBS, and dried at 37 °C; 33% glacial acetic acid (Sangon Biotech (Shanghai) Co., Ltd., A501931) was then added to dissolve the crystal violet, and 200 µl of the solution was taken to measure the absorbance at 590 nm on a spectrophotometer (Beckman, AD340). The degree of inhibition of biofilm formation ability by SSD was calculated by Formula (3):

3 $${\text{Percentage}}\;{\text{ of}}\;{\text{ inhibition}} = \left[ {1 - \frac{{{\text{OD }}\;{\text{value }}\;{\text{of }}\;{\text{experimental}}\;{\text{ group}} - {\text{OD }}\;{\text{value}}\;{\text{ of}}\;{\text{ blank }}\;{\text{control}}\;{\text{ group}}}}{{{\text{OD}}\;{\text{ value}}\;{\text{ of }}\;{\text{negative}}\;{\text{ group}} - {\text{OD }}\;{\text{value}}\;{\text{ of}}\;{\text{ blank}}\;{\text{ control}}\;{\text{ group}}}}} \right] \times 100{\text{\% }}$$

### XTT reduction assay

The cultured silicone membranes were washed three times with PBS and transferred into the wells of a 96-well deep-well plate, 500 µl of 2,3-bis(2-methoxy-4-nitro-5-sulfophenyl)-2H-tetrazolium-5-carboxanilide) (XTT) assay working solution (KeyGEN BioTECH, KGA313) was added, and the plate was incubated at 37 °C for 4 h in the dark. The absorbance at 450 nm was measured by a spectrophotometer. The degree of inhibition of biofilm metabolic activity by SSD was calculated using Formula (2).

### Scanning electron microscopy (SEM)

The cultured medical silicone membrane was rinsed three times with PBS, fixed overnight at 4 °C in 2.5% glutaraldehyde phosphate buffer (BBI, A600875), washed twice with 0.15% glutaraldehyde phosphate buffer, and then dehydrated with an ethanol series (40%, 70%, 90%, 100%) for 15 min with each concentration. Then, the cells were dried in a critical-point desiccator. After gold was sprayed with a vacuum coating device, the changes in the ultraspatial structure of the voice prosthesis biofilms in each group were observed under scanning electron microscopy (SEM) (ZEISS, GeminiSEM 360).

### Laser confocal microscopy

Live and dead bacteria on the biofilms were stained according to the instructions of the LIVE/DEAD BACLIGHT BACTERIAL C 1 KIT (Invitrogen, L7012). The culture medium was aspirated, and 0.85% NaCl was added to the well plate to rinse the medical silicone membranes three times. Equal volumes of component A (SYTO 9 dye, 3.34 mM) and component B (propidium iodide, 20 mM) were mixed thoroughly, and 3 µl of the mixed dye solution was added to each well. The plate was incubated for 15 min at room temperature in the dark. The distribution of live and dead bacteria in the biofilms in each group was observed under a laser confocal microscope (Olympus, FV3000).

### Polysaccharide‒phenol‒sulfuric acid method

The cultured medical silicone membranes were washed with PBS, transferred into a centrifuge tube, and 1 mm glass beads washed with concentrated hydrochloric acid were added. The samples were then placed into an automatic rapid sample grinder with 100 µl of PBS. The biofilms were eluted by shaking four times at 60 Hz for 60 s. A 5% phenol solution (Sinopharm Shanghai Test, 10015328) and 98% concentrated sulfuric acid (Sinopharm Shanghai Test, 10021608) were mixed to prepare the chromogenic solution at a ratio of 1:5. A total of 180 µl of the chromogenic solution was added to 60 µl different concentrations of glucose solution (Amresco, 0188) and the biofilm eluate. After thorough mixing, the mixture was heated in a metal bath at 100 °C for 25 min, after which 100 µl of the solution was removed to measure the absorbance at 490 nm on a spectrophotometer.

### Bicinchoninic acid (BCA) method

Protein determination was performed with the abovementioned eluates. CST RIPA buffer (Biyuntian, P0013B) was used to lyse each group of biofilms and collect proteins. Different concentrations of bovine serum albumin (BSA) standard and bicinchoninic acid (BCA) working solution were prepared according to the instructions of the BCA protein concentration determination kit (Sangon Biotech, C503021). Fifty microlitres of sample lysis buffer or 500 µl of BSA standard was mixed quickly with 500 µl of BCA working solution. The mixture was placed in a water bath at 37 °C for 30 min and then cooled to room temperature, and the A560 value was measured on a spectrophotometer.

### Effect of SSD on the mature biofilm on patients’ voice prostheses

To further verify the inhibitory and removal effects of SSD on mature biofilms on voice prostheses, we tested the effects on voice prosthesis biofilms from patients in vitro. We collected isolated, nonfunctional voice prostheses from three patients. For collection and sampling of patients’ voice prostheses, we obtained ethical certification and approval from the Ethics Committee of Peking Union Medical College Hospital (ethics number: JS2084). The biofilms on the voice prostheses were eluted by ultrasonication, and the bacterial strains in the biofilms were collected. The sterilized medical silicone membranes and 2 ml of the bacterial solution collected above were placed obliquely in the wells of a 96-well deep-well plate and incubated at 37 °C for 48 h. Different concentrations of SSD were added, with a medium control was established at the same time, and the cells were cultured at 37 °C for 24 h. The plate counting method was used to determine the effect of different concentrations of SSD on the number of bacterial colonies in the mature biofilms from the patients’ voice prostheses, and the XTT method was used to verify the inhibitory effect of SSD on the metabolic activity of the biofilms.

### Data analysis

Experiments in each group were repeated at least three times. The measurement data are expressed as the mean ± standard deviation. Differences among multiple groups were analysed by one-way analysis of variance (ANOVA). Comparison between the experimental group and the negative control group was performed by Dunnett’s t test. The data were analysed using GraphPad Prism 9 software. All tests were two-sided, and *P* < 0.05 indicated that the difference was statistically significant.

## Results

### Determination of the minimum BIC and the minimum BEC for mature biofilms on voice prostheses

**Fig. 1:**
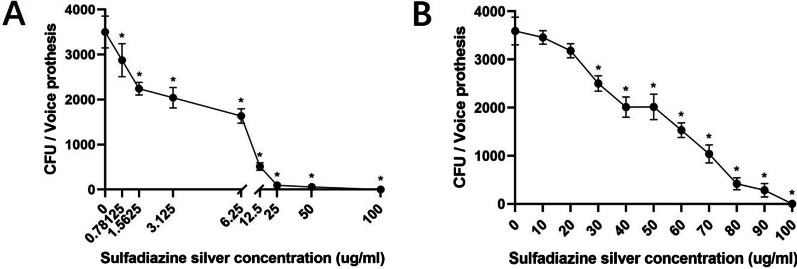
Effect of SSD on voice prosthesis biofilms. **A** Effect of SSD on the formation of voice prosthesis biofilms. **B** Effect of SSD on the number of colonies in mature mixed bacterial voice prosthesis biofilms. The experimental results are expressed as the mean ± SD. *P < 0.05 indicates a significant difference compared with the negative control group

The number of colonies in the mature mixed bacterial voice prosthesis biofilms was measured by the plate counting method to determine the minimum BIC and BEC of SSD. As shown in Fig. 1A, the number of colonies in the biofilms decreased gradually when the SSD concentration increased from 0 µg/ml to 6.25 µg/ml and decreased significantly as the SSD concentration increased from 6.25 to 100 µg/ml; at an SSD concentration of 6.25 µg/ml, the number of colonies in the biofilms decreased by 50%, i.e., the BIC_50_ was 6.25 µg/ml; at an SSD concentration of 100 µg/ml, no colonies were present in the biofilm. Hence, SSD had an inhibitory effect on biofilm formation on voice prostheses, and with increasing SSD concentration, the inhibitory effect was more obvious. As shown in Fig. 1B, the number of colonies in the biofilm of the SSD group was significantly reduced compared with that in the biofilm of the negative control group. Compared with the negative control group, the minimum SSD concentration for removing 30% of the mature biofilm, that is, the BEC_30_, was 30 µg/ml; the minimum SSD concentration for removing 50% of the mature biofilm (BEC_50_) was 50 µg/ml; the minimum SSD concentration for removing 70% of the mature biofilms (BEC_70_) was 70 µg/ml; and no colonies were found in the biofilm when the SSD concentration was 100 µg/ml, that is, the BEC_100_ was 100 µg/ml. Therefore, SSD could remove bacterial colonies in mature mixed bacterial voice prosthesis biofilms, and the removal effect became more obvious with increasing SSD concentration.

### Effect of SSD on mature mixed bacterial biofilms on voice prosthesis in an in vitro model

**Fig. 2:**
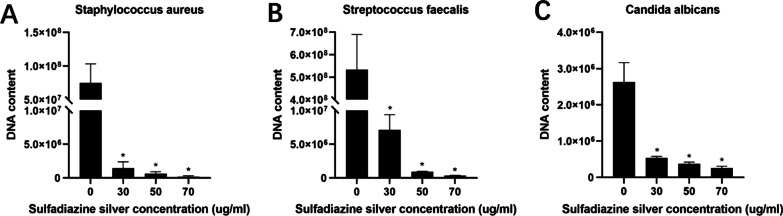
Effect of SSD on the DNA content of the three strains in mature mixed bacterial voice prosthesis biofilms. **A** Effect of SSD on *Staphylococcus aureus* in the biofilms; **B** Effect of SSD on *Streptococcus faecalis* in the biofilms; **C** Effect of SSD on *Candida albicans* in the biofilms. The results are expressed as the mean ± SD. *P < 0.05 indicates a significant difference compared with the negative control group

**Fig. 3:**
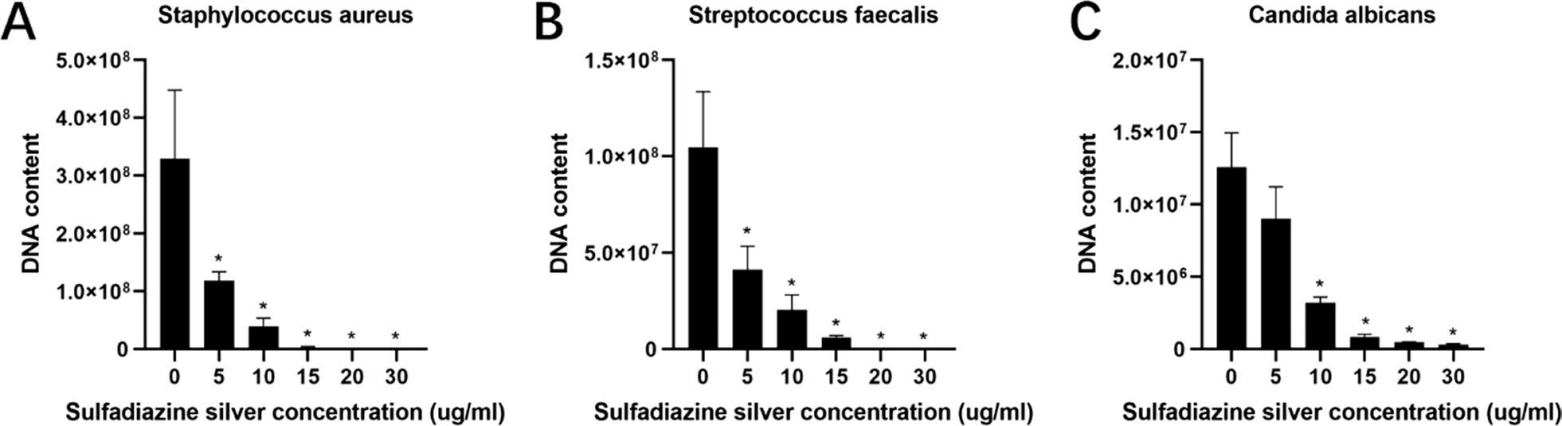
Effect of SSD at lower concentrations on the DNA content of the three strains in mature mixed bacterial voice prosthesis biofilms. **A** Effect of SSD on *Staphylococcus aureus* in the biofilms; **B** Effect of SSD on *Streptococcus faecalis* in the biofilms; **C** Effect of SSD on *Candida albicans* in the biofilms. The results are expressed as the mean ± SD. *P < 0.05 indicates a significant difference compared with the negative control group

RT‒qPCR was used to determine the copy numbers of *S. aureus*, *S. faecalis*, and *C. albicans* in mature mixed bacterial voice prosthesis biofilms in vitro to quantitatively study the effect of SSD on the removal of each of these three bacterial strains from voice prosthesis biofilms. In the experimental group, we first selected SSD concentrations of 30 µl/ml, 50 µl/ml and 70 µl/ml, i.e., the BEC_30_, BEC_50_, and BEC_70_, respectively. The results showed that the *S. aureus*, *S. faecalis*, and *C. albicans* DNA levels in the biofilms of the SSD group were significantly lower than those in the biofilms of the negative control group (Fig. 2), suggesting that SSD had significant inhibitory effects on the growth of the three bacteria. At an SSD concentration of 30 µl/ml, the number of bacterial colonies of these three bacterial strains was not significant. Therefore, to further study the effect of SSD concentration on the three bacterial strains in the mature voice prosthesis biofilms, we used SSD concentrations of 5 µg/ml, 10 µg/ml, 15 µg/ml, 20 µg/ml, and 30 µg/ml as the experimental group and repeated the above experiments. We found that SSD had inhibitory effects on the three bacteria in the biofilm at these lower concentrations. At the same concentration, SSD had the strongest inhibitory effect on *S. faecalis* among these three bacterial strains and the weakest inhibitory effect on *C. albicans*. The inhibitory effect against the three bacteria was particularly obvious, and at a concentration of 20 µg/ml, there were no distinct bacterial colonies (Fig. 3). To more intuitively show the inhibitory effect of SSD on the three bacteria and the associated trend, we selected three concentrations, 5 µg/ml, 10 µg/ml, and 15 µg/ml, for subsequent experiments.

The percentage reductions in copy number of the *S. aureus*, *S. faecalis*, and *C. albicans* in mature mixed bacterial voice prosthesis biofilms by different concentrations of SSD are shown in Table 2. SSD had an obvious removal effect on the three strains in mature mixed bacterial biofilms, and the higher the SSD concentration was, the more obvious the removal effect. The same concentration of SSD had a significant removal effect on *S. faecalis* and *S. aureus*, while the removal effect on *C. albicans* was relatively weak. At an SSD concentration of 20 µg/ml, the abundances of *S. faecalis* and *S. aureus* were reduced by more than 99%, and that of *C. albicans* was reduced by more than 95%.

**Table 2:** Percentage reductions in the *Staphylococcus aureus*, *Streptococcus faecalis* and *Candida albicans* DNA levels in mature mixed bacterial voice prosthesis biofilms by SSD (mean ± SD)

Strains	Groups				
	5 µg/ml SSD	10 µg/ml SSD	15 µg/ml SSD	20 µg/ml SSD	30 µg/ml SSD
*S. aureus*	52.61 ± 36.02%	76.02 ± 20.48%	93.50 ± 2.98%	99.66 ± 0.32%	99.93 ± 0.05%
*S. faecalis*	58.93 ± 17.90%	87.51 ± 5.01%	99.10 ± 0.50%	99.78 ± 0.15%	99.91 ± 0.06%
*C. albicans*	21.25 ± 46.14%	74.10 ± 2.54%	92.68 ± 3.38%	95.89 ± 1.02%	97.34 ± 1.01%

**Fig. 4:**
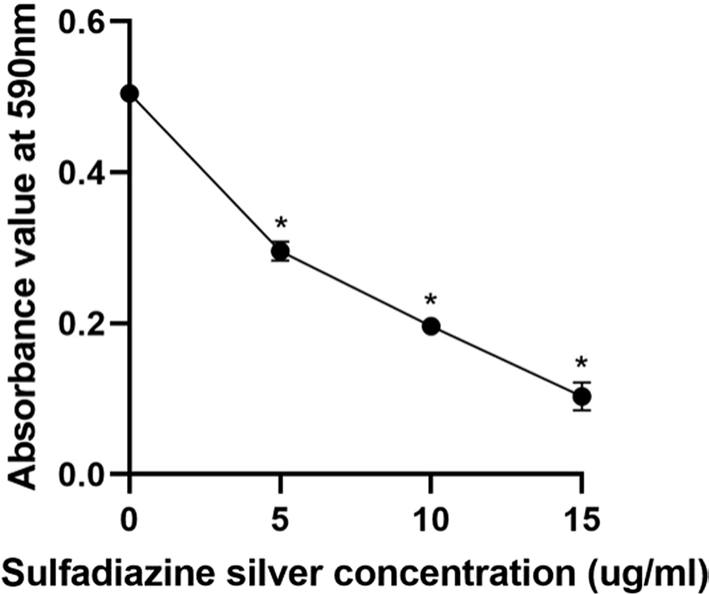
Effect of SSD on the biofilm formation ability of the mature mixed strains on voice prostheses. The experimental results are expressed as the mean ± SD. *P < 0.05 indicates a significant difference compared with the negative control group

Crystal violet staining was used to determine the effect of SSD on the biofilm formation ability of the mature mixed strains on voice prostheses. Compared with the negative control group, SSD significantly reduced the absorbance of the stained biofilms at 590 nm, and the higher the concentration of SSD was, the more obvious the decrease in absorbance (Fig. 4), suggesting that SSD could significantly inhibit the biofilm formation ability of the mature mixed strains on voice prostheses.

**Fig. 5:**
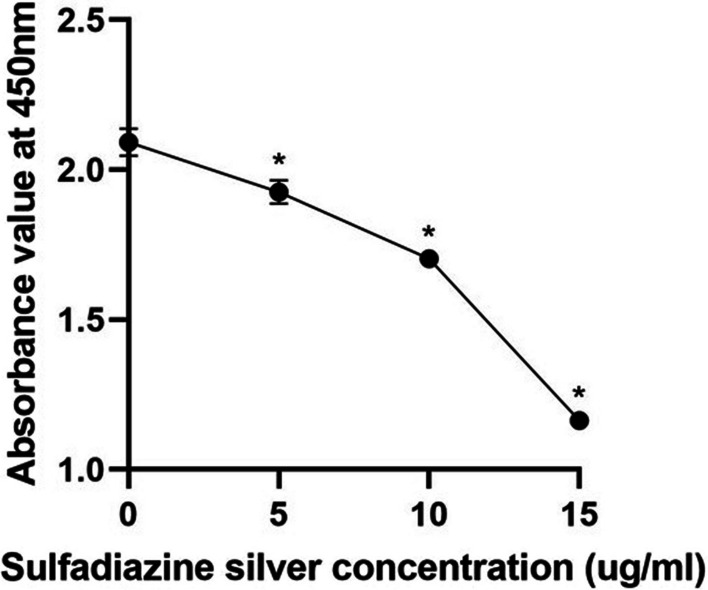
Effect of SSD on the biofilm metabolic activity of the mature mixed voice prosthesis strains. The experimental results are expressed as the mean ± SD. *P < 0.05 indicates a significant difference compared with the negative control group

An XTT reduction assay was used to measure the absorbance of the mature mixed bacterial voice prosthesis biofilms at 450 nm. The results showed that SSD significantly inhibited the absorbance of the biofilms at 450 nm, and the inhibition became more significant with increasing SSD concentration (Fig. 5), suggesting that SSD could significantly inhibit the metabolic activity of mature voice prosthesis biofilms.

The percentage reductions caused by different concentrations of SSD on the biofilm formation ability and metabolic activity of the mature mixed strains on voice prostheses are shown in Table 3. The degree of inhibition of the biofilm formation ability and metabolic activity of the mature mixed strains on voice prostheses increased with increasing SSD concentration.

**Table 3:** Percentage reductions in the biofilm formation ability and metabolic activity of mature mixed strains on voice prostheses caused by SSD (mean ± SD)

Experiments	Groups		
	5 µg/ml SSD	10 µg/ml SSD	15 µg/ml SSD
Biofilm formation ability	45.46 ± 3.89%	67.24 ± 1.63%	87.49 ± 4.40%
Biofilm metabolic activity	13.97 ± 2.39%	32.68 ± 1.28%	79.05 ± 5.59%

**Fig. 6:**
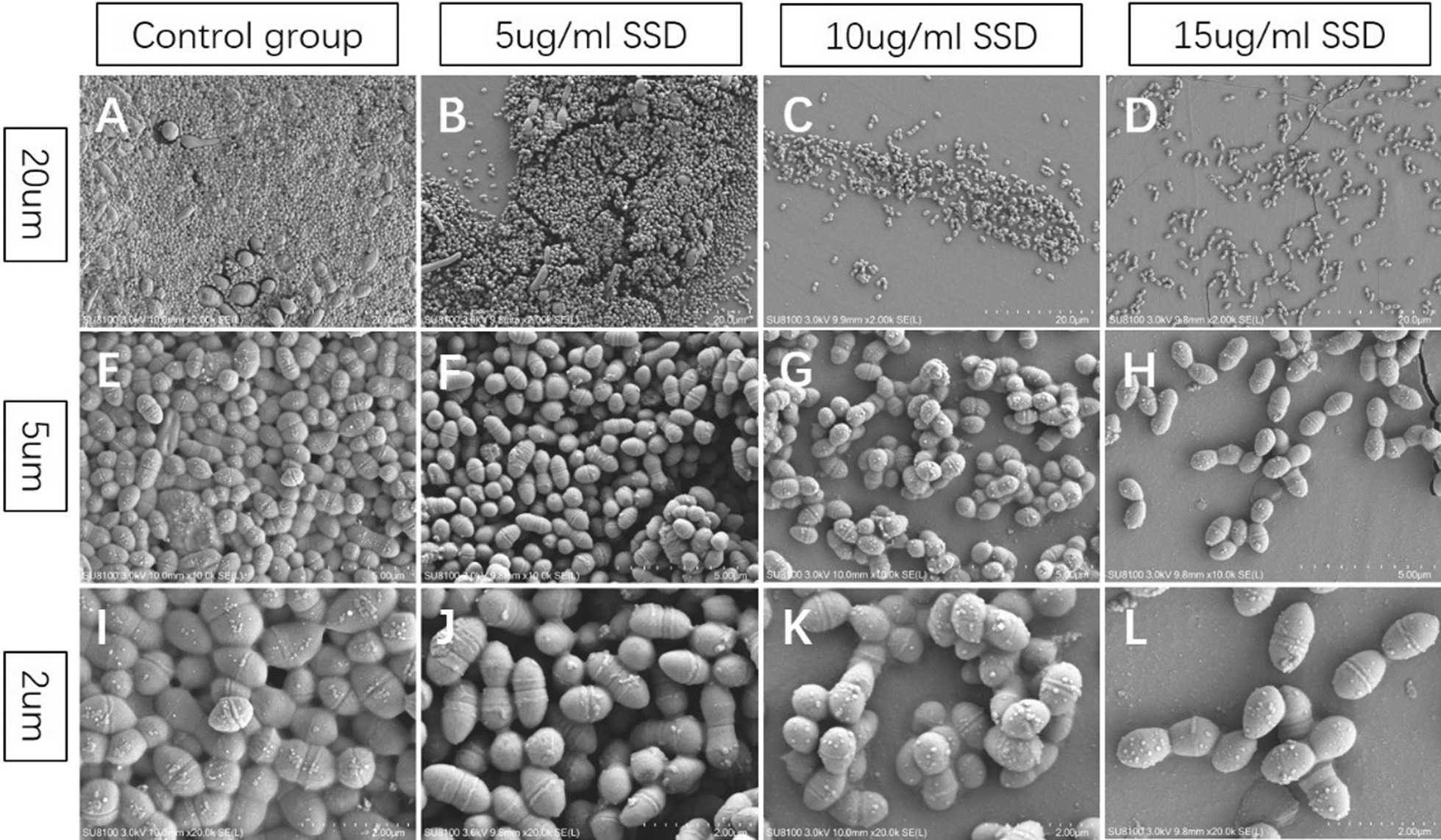
Ultrastructure of the voice prosthesis biofilms by SSD

SEM was used to observe the ultrastructure of the mature voice prosthesis biofilms (Fig. 6). We found that in the negative control group, most of the medical silicone membrane was covered by the biofilm, and the colonies in the biofilm were densely arranged, forming a complex spatial structure (Fig. 6A, E, I). When the SSD concentration was 5 µg/ml, the area covered by the biofilm on the medical silicone membrane and the number of colonies in the biofilm were significantly reduced, and the tightness of the arrangement between the strains was decreased (Fig. 6B, F, J). When the SSD concentration was 10 µg/ml, the area covered by biofilms and the number of colonies were further reduced. Although a small number of strains aggregated to form colonies, the colonies were scattered, and no biofilms with complex spatial structures were found (Fig. 6C, G, K). When the SSD concentration was 15 µg/ml, the strains on the medical silicone membrane were scattered, and there was no obvious biofilm or colony formation (Fig. 6D, H, L). SEM clearly revealed that SSD could significantly remove strains and colonies in biofilms and inhibit the formation of complex spatial biofilm structures, with the inhibition and removal of biofilms becoming more obvious with increasing SSD concentration.

**Fig. 7:**
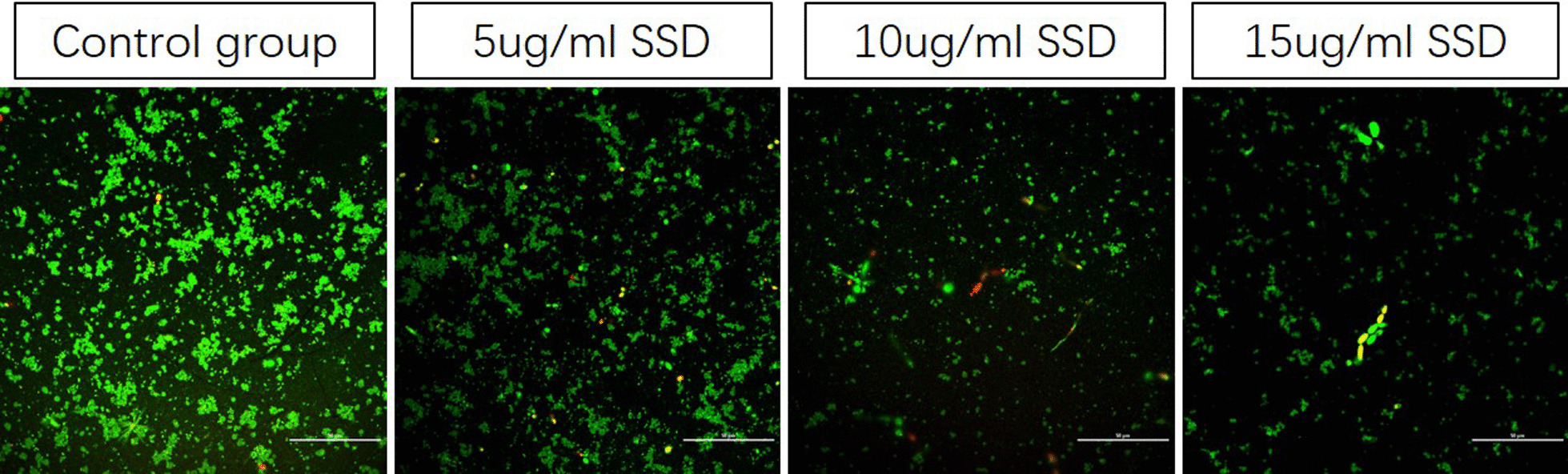
Distribution of live and dead bacteria on the mature mixed bacterial voice prosthesis biofilms at different concentrations of SSD. In the figure, green fluorescence indicates live bacteria, and red fluorescence indicates dead bacteria

In addition, we observed the effect of different concentrations of SSD on the distribution of live and dead bacteria in mature mixed bacterial voice prosthesis biofilms by laser confocal microscopy. As shown in Fig. 7, a large number of viable bacterial colonies could be seen in the biofilm of the negative control group. Compared with the negative control group, when the SSD concentration was 5 µg/ml, the number of colonies in the biofilm was significantly reduced, and most of the strains in the biofilm were live bacteria; when the SSD concentration was 10 µg/ml, the number of colonies in the biofilm was further decreased. Live and dead bacteria were observed in the biofilm colonies. When the concentration of SSD was 15 µg/ml, only a small number of colonies remained in the biofilm. These results suggest that SSD could effectively remove and kill the strains in mature mixed bacterial voice prosthesis biofilms, and the higher the concentration was, the more significant the effect.

**Fig. 8:**
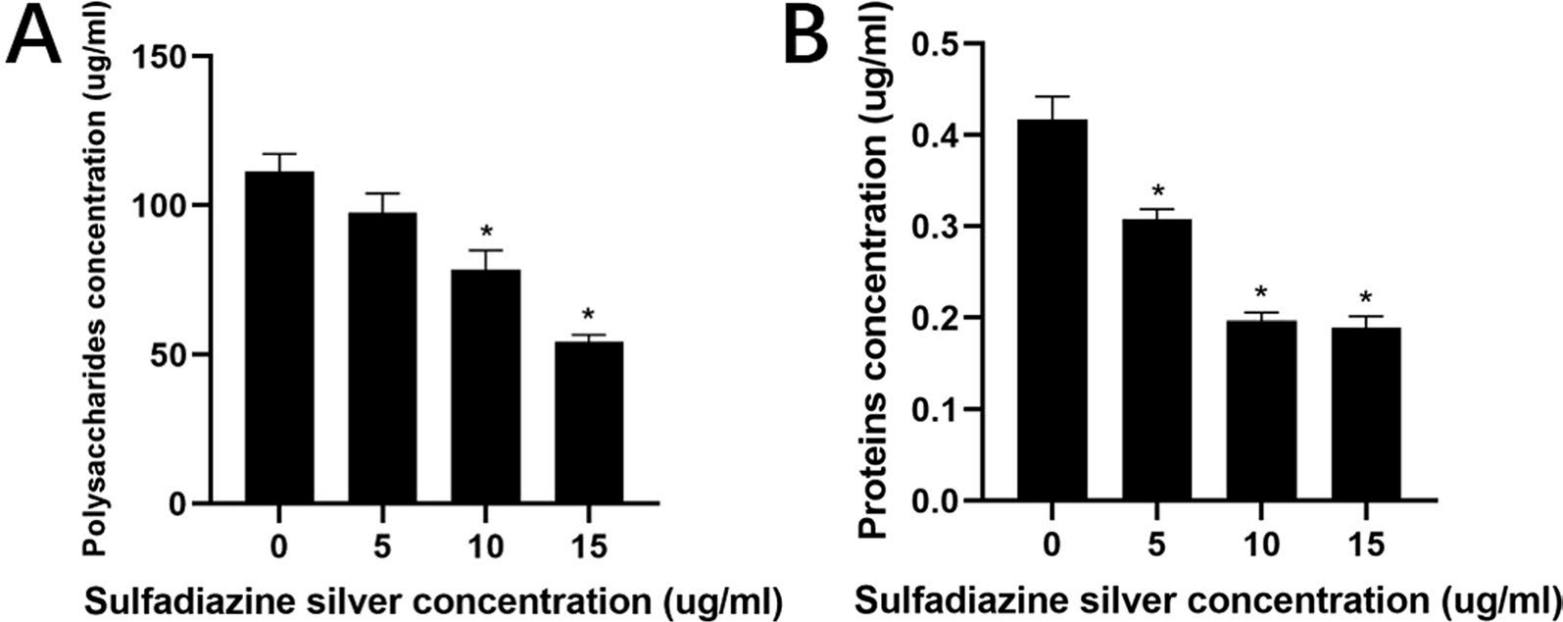
Effects of SSD on polysaccharides (**A**) and proteins (**B**) in mature mixed bacterial voice prosthesis biofilms. The experimental results are expressed as the mean ± SD. *P < 0.05 indicates a significant difference compared with the negative control group

The polysaccharide and protein levels in the biofilms were determined by the polysaccharide‒phenol‒sulfuric acid and BCA methods, respectively. Compared with those in the negative control group, the polysaccharide and protein levels in the biofilms gradually decreased with increasing SSD concentration (Fig. 8). When the SSD concentration was 10 µg/ml, the polysaccharide content in the biofilm decreased significantly (Fig. 8A, *P* < 0.05); when the SSD concentration was 5 µg/ml, the protein content in the biofilm decreased significantly (Fig. 8B, *P* < 0.05). These results suggest that SSD could inhibit the synthesis of polysaccharides and proteins in the biofilm extracellular matrix.

### Effect of SSD on mature mixed bacterial voice prosthesis biofilms in patients

**Fig. 9:**
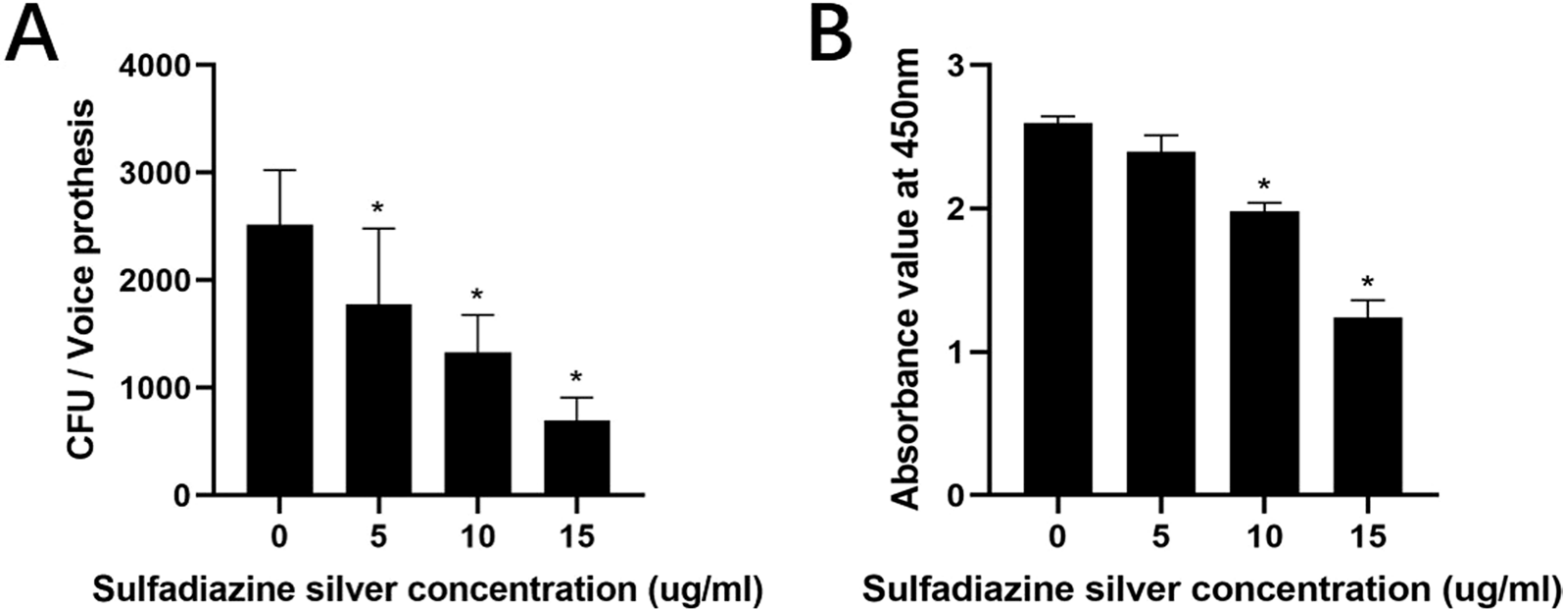
Effects of SSD on the number of colonies (**A**) and the metabolic activity of the strains (**B**) in the patients’ mature mixed voice prosthesis biofilms. The experimental results are expressed as the mean ± SD. *P < 0.05 indicates a significant difference compared with the negative control group

The plate counting method and XTT reduction assay were used to verify the inhibition and removal effect of SSD on mature mixed bacterial voice prosthesis biofilms from patients. Compared with the negative control group, the number of biofilm colonies and the absorbance value of the biofilms in the SSD groups decreased significantly (Fig. 9, *P* < 0.05) with increasing SSD concentration. The percentage reduction caused by SSD in the number of mature biofilm colonies and the metabolic activity of the biofilms on the patients’ voice prostheses are shown in Table 4. The results suggest that SSD also had inhibitory and removal effects on the mature biofilms on the patients’ voice prostheses, and when the SSD concentration was 5 µg/ml, a significant reduction in the number of bacterial colonies in the biofilms was observed. The metabolic activity of the mature biofilms was significantly reduced when the SSD concentration was 10 µg/ml.

**Table 4:** Percentage reductions in the number of colonies and metabolic activity of mature mixed bacterial voice prosthesis biofilms caused by SSD in patients (mean ± SD)

Experiments	Groups		
	5 µg/ml SSD	10 µg/ml SSD	15 µg/ml SSD
Number of biofilm colonies	24.81 ± 14.47%	42.21 ± 9.26%	72.28 ± 2.09%
Biofilm metabolic activity	9.29 ± 6.46%	28.82 ± 4.44%	63.36 ± 4.14%

## Discussion

Biofilms are aggregates of microorganisms composed of dense layers of microorganisms and extracellular polymeric substances (EPS) [22]. Biofilms not only provide sites for microbial colonization but also protect the microbes against antimicrobial agents [23]. Although the excellent mechanical and moulding properties of silicone make it the best material for voice prostheses, due to the hydrophobicity of silicone, coupled with the continuous exposure of voice prostheses to saliva, food, and oropharyngeal flora [24], bacteria and yeasts colonize this material readily and quickly, forming a biofilm [25], which in turn destroys the function of the voice prosthesis and reduces its lifespan. However, frequent replacement of voice prostheses imposes great burden on patients physiologically, psychologically, and financially. Thus, a method for inhibiting or removing voice prosthesis biofilms to prolong the lifespan of the device is urgently needed.

Many scholars have carried out research on inhibiting the formation of voice prosthesis biofilms, but the results have confirmed that due to the existence of drug resistance, antifungal and antibacterial drugs cannot effectively inhibit the formation of these biofilms [26, 27]. Therefore, researchers hope to prevent biofilm formation on biological materials by enhancing the anticontamination properties of the silicone surface of voice prostheses, thereby prolonging their lifespan. Related studies have included the use of metal nanoparticle coatings, chitosan coatings, biosurfactants, essential oil coatings, and laser grafting of hydrophilic monomers [28–32]. Although these methods can effectively inhibit biofilm formation on voice prostheses in vitro, considering the complexity of the in vivo environment, these methods cannot effectively prolong the lifespan of voice prostheses, mainly because the active surface becomes covered with proteins and necrotic cells, thus inhibiting their anticontamination effects [33]. Therefore, to date, there is no effective method for inhibiting voice prosthesis biofilm formation in clinical practice. An alternative strategy is to prolong the lifespan of the voice prosthesis by removing the mature biofilm from the voice prosthesis. In this study, the number of colonies, biofilm formation ability, metabolic activity, and ultrastructure of mature mixed bacterial voice prosthesis biofilms under SSD treatment were studied in vitro to explore the inhibition and removal of biofilms by SSD. Our study showed that SSD could inhibit and remove mature mixed bacterial voice prosthesis biofilms, and the higher the SSD concentration was, the more significant the inhibition and removal; when the SSD concentration was 5 µg/ml, the removal effect on biofilm colonies was significant. When the SSD concentration was 20 µg/ml, there was no noticeable flora in the mature mixed bacterial voice prosthesis biofilms. Moreover, we further confirmed our hypothesis in vitro on the voice prosthesis biofilms of patients and obtained consistent results.

As important components of biofilms, EPS have received increasing attention. EPS are generally composed of water, extracellular polysaccharides, extracellular proteins, extracellular deoxyribonucleic acid (eDNA), and lipids [34]. Studies have shown that extracellular polysaccharides endow bacteria and EPS with adhesion properties, provide shape and structural support for biofilms [35], hinder the penetration of antimicrobial drugs into biofilms and enhance drug resistance [36]. Extracellular proteins act as “adhesion polymers”, forming the pericellular microenvironment and participating in the maintenance of biofilm integrity and stability [37, 38]. In the present study, we also examined polysaccharides and proteins in mature mixed bacterial voice prosthesis biofilms. Our results showed that the polysaccharide and protein levels in the biofilms of the SSD groups were significantly lower than those in the biofilms of the negative control group, and the biofilm amount, biofilm formation ability, and metabolic activity of the biofilms in the SSD groups were also significantly lower than those in the negative control group. Combined with the SEM results, these findings indicate that SSD inhibited the formation of the complex spatial structure of voice prosthesis biofilms. We speculate that SSD may destroy the integrity and stability of voice prosthesis biofilms by inhibiting polysaccharide and protein synthesis in the EPS of the biofilm, making it easier for SSD to penetrate and diffuse into the biofilm, thereby reducing the biological resistance of the biofilms to SSD and ultimately exerting a significant inhibitory and removal effect on mature voice prosthesis biofilms.

Our study shows that SSD has the potential to inhibit and remove mature mixed bacterial voice prosthesis biofilms, providing a novel idea for prolongation of the lifespan of voice prostheses. Of course, further in-depth research on the mechanism underlying the effect of SSD on biofilms is needed, with further support from in vivo experiments to provide a basis for the clinical treatment of mature voice prosthesis biofilms by SSD.

## Conclusions

SSD could effectively inhibit and remove mature mixed bacterial voice prosthesis biofilms, reduce biofilm formation ability and metabolic activity. The higher the concentration of SSD was, the more obvious the inhibition and removal effects on the biofilms. SSD may act by inhibiting the synthesis of polysaccharides and proteins in the EPS of voice prosthesis biofilms, thus disrupting the integrity and stability of voice prosthesis biofilms. Our results provide a novel basis for the treatment of patients’ mature voice prosthesis biofilms which has clinical potential in the future.

## Data Availability

All data generated or analysed during this study are included in this published article.

## Abbreviations

SSD: Silver sulfadiazine
XTT: 2,3-Bis(2-methoxy-4-nitro-5-sulfophenyl)-2H-tetrazolium-5-carboxanilide
YPDF: Yeast extract/peptone/dextrose–fetal bovine serum
BIC: Biofilm inhibitory concentration
BEC: Biofilm eradication concentration
PBS: Phosphate-buffered saline
SEM: Scanning electron microscopy
BSA: Bovine serum albumin
BCA: Bicinchoninic acid
EPS: Extracellular polymeric substances
